# Benchmarking datasets for assembly-based variant calling using high-fidelity long reads

**DOI:** 10.1186/s12864-023-09255-y

**Published:** 2023-03-27

**Authors:** Hyunji Lee, Jun Kim, Junho Lee

**Affiliations:** 1 Institute of Molecular Biology and Genetics, Seoul National University, Seoul, 08826 Korea; 2 Department of Biological Sciences, Seoul National University, Seoul, 08826 Korea; 3 Research Institute of Basic Sciences, Seoul National University, Seoul, 08826 Korea; 4 Department of Convergent Bioscience and Informatics, College of Bioscience and Biotechnology, Chungnam National University, Daejeon, 34134 Korea

**Keywords:** Genetic variant, Variant calling, High-fidelity long reads, Long-read sequencing, Benchmark

## Abstract

**Background:**

Recent advances in long-read sequencing technologies have enabled accurate identification of all genetic variants in individuals or cells; this procedure is known as variant calling. However, benchmarking studies on variant calling using different long-read sequencing technologies are still lacking.

**Results:**

We used two *Caenorhabditis elegans* strains to measure several variant calling metrics. These two strains shared true-positive genetic variants that were introduced during strain generation. In addition, both strains contained common and distinguishable variants induced by DNA damage, possibly leading to false-positive estimation. We obtained accurate and noisy long reads from both strains using high-fidelity (HiFi) and continuous long-read (CLR) sequencing platforms, and compared the variant calling performance of the two platforms. HiFi identified a 1.65-fold higher number of true-positive variants on average, with 60% fewer false-positive variants, than CLR did. We also compared read-based and assembly-based variant calling methods in combination with subsampling of various sequencing depths and demonstrated that variant calling after genome assembly was particularly effective for detection of large insertions, even with 10 × sequencing depth of accurate long-read sequencing data.

**Conclusions:**

By directly comparing the two long-read sequencing technologies, we demonstrated that variant calling after genome assembly with 10 × or more depth of accurate long-read sequencing data allowed reliable detection of true-positive variants. Considering the high cost of HiFi sequencing, we herein propose appropriate methodologies for performing cost-effective and high-quality variant calling: 10 × assembly-based variant calling. The results of the present study may facilitate the development of methods for identifying all genetic variants at the population level.

**Supplementary Information:**

The online version contains supplementary material available at 10.1186/s12864-023-09255-y.

## Background

Accurate variant detection is required for reliable assessments of the relationships between genotypes and phenotypes. Several population-level studies have elucidated the genetic architectures responsible for a range of quantitative traits, including susceptibility to common human diseases [1], inherited human diseases [2, 3], and crop optimization in plants [4], using short-read sequencing technologies owing to their high throughput and low cost [5]. However, short-read sequencing is inadequate for accurately identifying large structural variants (SVs) due to the short length of reads [6]. In particular, short-read-based methods employed for detecting variants by mapping reads to reference genomes commonly miss variants not existing in the reference genomes, such as insertions [7]. Thus, these methods have limited utility in determining the precise genetic sequences underlying quantitative traits [8].

In recent years, long-read sequencing techniques and related computational tools have significantly overcome these limitations [9, 10]. Long-read sequencing is a method that can read nucleotide sequences ranging from tens of kilobases (kb) to several megabases (Mb) at a time [11]. Although long-read sequencing is typically less accurate than short-read sequencing, it allows identification of SVs including insertions due to the long length of reads [12]. Furthermore, the limitation of low throughput of long-read sequencing is gradually being tackled with the use of the Sequel II system from Pacific Biosciences (PacBio) and PromethION system from Oxford Nanopore Technologies [13]. These systems provide high throughput, and their costs are still much more expensive, but become similar to those of short-read sequencing technologies [13]. Moreover, the high-fidelity (HiFi) long-read sequencing technology developed by PacBio has been shown to improve base-level accuracy by up to 99.9% by utilizing circular consensus sequences of 10–20 kb [14]. Compared with the conventional noisy continuous long-read (CLR) sequencing technology, HiFi sequencing allows rapid assembly of high-quality genomes and requires few computational resources due to its reliable base quality [14, 15].

By applying the advantages of long-read sequencing technologies in terms of detecting SVs and generating high-quality genome assemblies, raw read-based and genome assembly-based variant detection methodologies are rapidly being improved [16, 17]. Although 100-fold longer reads may allow detection of previously unidentified SVs, the use of calling variants exceeding the read length remains technically challenging [18]. This limitation can be overcome by assembling long reads into large contigs using the overlap between reads, which are 10- to 100-fold longer than the reads [13]. As contigs have comparable lengths to chromosomes, they can be used to accurately identify all genetic variants in a population, even at the chromosomal level [19–22]. However, as high-quality genome assembly requires 20 × sequence depth and read-based variant calling requires 5 × sequence depth, systematic benchmarking analysis is required to identify genetic variants in an accurate and cost-effective manner [23, 24].

Herein, we used a benchmarking dataset to compare the variant calling performance of accurate and noisy long-read sequencing technologies. Using this dataset, we estimated the relative true- and false-positive ratios as well as read- and assembly-based true-positive detection rates at various depths. Our results may provide a basis for future large-scale pangenome studies to elucidate genetic variants in any given population.

## Results

### Experimental design for variant detection analysis in *Caenorhabditis elegans*

**Fig. 1:**
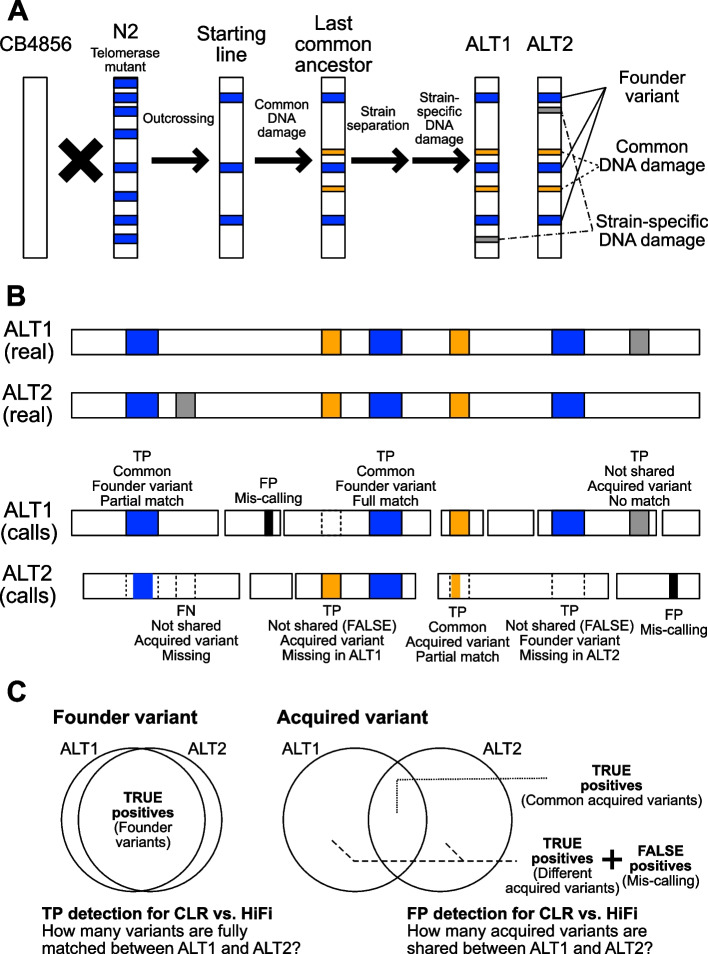
Experimental scheme for the generation of *C*. *elegans* strains to estimate variant calling performance. **A** Schematic representation of genetic variants in the two *C*. *elegans* strains. Two strains, namely ALT1 and ALT2, were derived from a common telomerase mutant worm. When CB4856 genome was used as a reference, all variants detected in the ALT1 and ALT2 assemblies can be divided into the following three types: (1) founder variants introduced during the process of starting mutant worm generation (blue bars); (2) variants acquired by common DNA damage events (yellow bars); and (3) variants acquired by strain-specific DNA damage events (gray bars). The location of each variant does not reflect the real location. **B** Comparison of various types of real variants and variants that were identified by sequencing and/or variant calling errors. Identified variants can be categorized as follows: (1) full match: if the identified variant exactly matched the corresponding real variant; (2) partial match: if the identified variant partially matched the corresponding real variant; (3) missing: if the real variant existed in the genome but no corresponding variant was identified; and (4) mis-calling: no real variant existed at a certain locus but the locus contained an identified variant; TP, true positive; FP, false positive. **C** Types of variants used for estimating the variant calling performance of CLR and HiFi. Founder variants can be accurately identified by utilizing the high-quality N2 genome. The number of shared founder variants between ALT1 and ALT2 was used to measure the true-positive detection performance for HiFi and CLR (left). The two strains contained true-positive variants acquired through common and strain-specific DNA damage in addition to falsely detected miscalled variants. The ratio of common acquired variants and strain-specific acquired variants was utilized to measure the false-positive detection performance for HiFi and CLR (right)

We used two *C. elegans* strains to compare the variant calling performance of accurate (HiFi) and noisy (CLR) long-read sequencing technologies. Two strains, namely, ALT1 and ALT2, were generated from a common telomerase mutant worm (Fig. 1A). This telomerase mutant worm had a predominant CB4856-type genetic background and contained some genomic segments from the founder strain N2, which were introduced during the process of mutant generation. Then, telomere damage-mediated mutations were introduced and descendants were separated into two lines after sufficient DNA damage and chromosome fusion events. The genomic sequences of the obtained two lines were identical at the time of generation but diverged later due to line-specific DNA damage prior to telomere stabilization, thereby leaving strain-specific mutations. Thus, we used the CB4856 genome as a reference. All variants detected in the ALT1 and ALT2 assemblies were divided into the following two types: (1) variants shared with the founder, hereafter named “founder variants” (overlap with known N2 variants) and (2) variants acquired by common or strain-specific DNA damage events, hereafter named “acquired variants” (no overlap with known N2 variants; Fig. 1A).

We used variation characteristics to compare the true-positive and false-positive ratios of HiFi and CLR because all true-positive variants in ALT1 and ALT2 were not known. Since sequencing, genome assembly, and variant calling processes are not perfect, some identified variants may not be identical to their corresponding real variants in the genome. Thus, we categorized the identified variants as follows: (1) full match: if the identified variant exactly matched a real variant, i.e., a true-positive variant; (2) partial match: if the identified variant partially matched a real variant; (3) missing: if the real variant existed in the genome but no corresponding variant was identified; and (4) mis-calling: no real variant existed at a certain locus but a corresponding variant was identified (Fig. 1B).

Moreover, we utilized the known characteristics of the founder and acquired variants. Founder variants were introduced during the generation of the starting line. Accordingly, most if not all of the founder variants were assumed to be shared among the three strains, i.e., ALT1, ALT2, and the founder N2, unless they were lost or altered due to mutations caused by strain-specific DNA damage. Due to the availability of a high-quality, long-read sequencing-based N2 genome, we were able to distinguish founder variants from other variants in the ALT1 and ALT2 genomes. We used these founder variants to measure the relative performance of HiFi and CLR in detecting true-positive variants by calculating the ratio of common to all founder variant numbers. A higher number of shared founder variants between ALT1 and ALT2 indicated a higher true-positive detection performance for a given technology (Fig. 1C).

Furthermore, the two strains used in the present study had common and strain-specific DNA damage-mediated variants that were acquired before and after line separation, respectively (Fig. 1C). Accordingly, variants shared between the two strains were assumed to be true-positive variants formed as a result of common DNA damage. In contrast, the variants that were not shared between the strains were assumed to be true-positive strain-specific variants or falsely detected miscalled variants. Using these acquired variant datasets, we estimated the relative performance of HiFi and CLR in detecting false-positive variants. We assumed that the true ratio of strain-specific to common acquired variants of the two strains should be the same regardless of the sequencing technologies and that the ratio of HiFi that we detected can be considered as a proxy of the true ratio. Based on the ratio of HiFi, we suspected that the remaining number of strain-specific acquired variants detected in CLR would be false-positives.

### HiFi assemblies showed two-fold higher contiguity than depth-matched CLR assemblies

**Fig. 2:**
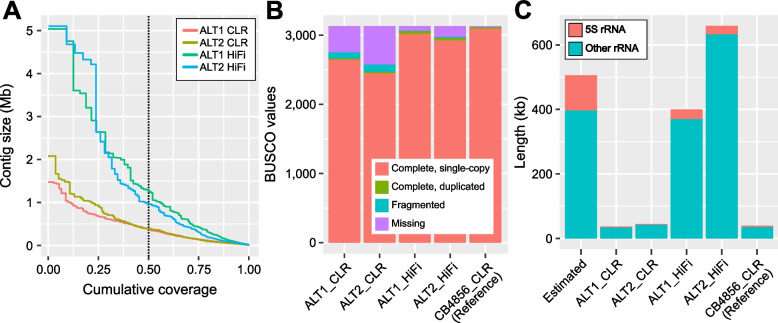
HiFi assemblies showed twofold higher contiguity than depth-matched CLR assemblies. **A** Nx plot showing the distribution of contig lengths in each assembly. The intersection of each horizontal solid line and vertical dotted line indicates the N50 of each assembly. **B** BUSCO values of each assembly. Note that the reference genome CB4856 was also built on CLR rather than HiFi. **C** Lengths of the ribosomal RNA sequences in each assembly and their previously estimated lengths in *C*. *elegans*

The two DNA samples were multiplexed for cost-effective HiFi sequencing, and 2.2-Gb and 1.9-Gb HiFi reads were generated from ALT1 and ALT2, respectively, after demultiplexing (Additional file 1: Table S1–2 and Fig. S1). The sequencing depth of ALT1 and ALT2 was approximately 20 × , which was comparable to that obtained using CLR [20]. We compared depth-matched HiFi and CLR assemblies and confirmed that HiFi assemblies showed higher contiguity than CLR assemblies. The N50 lengths of the ALT1 and ALT2 HiFi assemblies were 1.2 Mb and 1 Mb, respectively, and the maximum contig lengths were 5.0 Mb and 5.1 Mb, respectively, all of which were more than 2.5-fold higher than those of the depth-matched CLR assemblies (Fig. 2A and Additional file 1: Table S3). To further analyze assembly contiguity, we used BUSCO, which evaluates the completeness of single-copy ortholog genes in a given assembly, and confirmed that the number of fragmented or missing orthologs was reduced by approximately fivefold in the HiFi assembly compared with the CLR assembly (Fig. 2B). Finally, we evaluated the length of the ribosomal RNA clusters in each assembly as most ribosomal RNA clusters are disassembled due to their long and tandemly repeating units. The lengths of ribosomal RNA sequences in the HiFi-based genome assemblies were much closer to the previously estimated length in the *C*. *elegans* reference genome than in the CLR-based genome assemblies (Fig. 2C) [25]. These results demonstrate that HiFi outperforms CLR in terms of assembly contiguity.

### HiFi detected 37% more founder variants than CLR

Next, we analyzed the variant calling performance of HiFi and CLR assemblies. We used the CB4856 genome as a reference and called all types of variants ≥ 5 bp. The total number of variants (4405 for CLR and 3938 for HiFi in ALT1 and 3619 for CLR and 3678 for HiFi in ALT2) and overall length distribution of the variants (Average lengths: 180 bp for CLR and 258 bp for HiFi in ALT1 and 186 bp for CLR and 268 bp for HiFi in ALT2) were comparable between CLR and HiFi for both strains (Additional file 1: Fig. S2A–D).

We categorized ≥ 5-bp insertions and deletions into founder or acquired variants and further classified them according to their size (5–49 bp or ≥ 50 bp) and type of mutation (insertion or deletion). In both strains, HiFi identified more founder variants and a comparable number of total variants than CLR did (Additional file 1: Table S4). In the CLR assembly, 1719 and 1412 founder variants were detected in ALT1 and ALT2, respectively, whereas in the HiFi assembly, 2211 and 2066 founder variants were detected in ALT1 and ALT2, respectively (Additional file 1: Table S4). The difference between HiFi and CLR in detecting founder variants was more pronounced with larger variants. For 5–49-bp variants, HiFi detected 29% more founder variants than CLR (1135 for CLR vs. 1372 for HiFi in ALT1 and 918 for CLR vs. 1271 for HiFi in ALT2), and for ≥ 50-bp variants, HiFi detected 52% more founder variants than CLR (584 for CLR vs. 839 for HiFi in ALT1 and 494 for CLR vs. 795 for HiFi in ALT2) (Additional file 1: Table S4).

### HiFi outperformed CLR in detecting potential true-positive variants of 5–49 bp by 1.9-fold

**Fig. 3:**
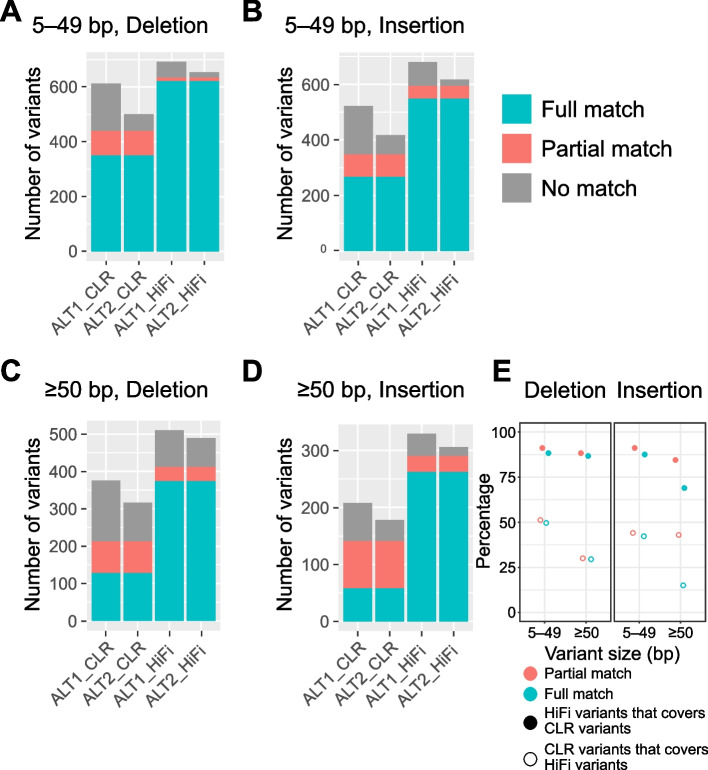
Founder variant detection rates of CLR and HiFi assemblies to estimate true-positive calling performance. **A**–**D**, Number of shared or unmatched founder variants detected in each assembly. Founder variants were divided into four groups according to their size and type: **A** 5–49-bp deletion; **B** 5–49-bp insertion; **C** ≥ 50-bp deletion; and **D** ≥ 50-bp insertion. Blue bar represents founder variants perfectly matched for position and size between ALT1 and ALT2 using the same sequencing mode. Red bar represents partially matched founder variants. Gray bar represents the remaining founder variants identified in a single strain. **E** Overlap ratios for common founder variants detected using CLR and HiFi assemblies. Full circle represents the ratio of CLR variants covered by HiFi and the empty circle represents vice versa. Colors represent partial- or full-matched variant sets

We next analyzed the true-positive detection performance of HiFi and CLR using the founder variants that were shared between ALT1 and ALT2. For 5–49-bp deletions, CLR detected 613 founder variants in ALT1 and 501 founder variants in ALT2. We identified only 350 perfectly matched (57% in ALT1 and 70% in ALT2) and 90 partially matched deletions (15% in ALT1 and 18% in ALT2) between ALT1 and ALT2 in terms of position and size. The remaining 173 and 61 deletions in ALT1 and ALT2, respectively, were strain-specific (Fig. 3A).

In contrast, HiFi detected 621 perfectly matched 5–49-bp founder deletions (90% in ALT1 and 95% in ALT2) between ALT1 and ALT2. The proportions of partially matched and strain-specific deletions were < 10%, being much lower than those detected using CLR (Fig. 3A). For 5–49-bp insertions, HiFi had a higher performance than CLR in detecting common founder variants between the two strains. However, despite the high performance, the proportion of 5–49-bp insertions that partially matched between the two strains was slightly higher than that of 5–49-bp deletions, indicating that the detection accuracy for 5–49-bp insertions was lower than that for similar-sized deletions (Fig. 3B).

When common founder variants that perfectly matched between ALT1 and ALT2 were considered as the true-positive variant set, the use of the HiFi assembly resulted in significantly improved true-positive variant calling performance, enabling detection of a 1.8-fold higher number of 5–49-bp deletions and a two-fold higher number of 5–49-bp insertions than those identified using the CLR assembly. Moreover, when common founder variants that at least partially matched between the two strains were considered, the use of the HiFi assembly allowed the detection of a 1.4-fold higher number of 5–49-bp deletions and 1.7-fold higher number of 5–49-bp insertions than the CLR assembly.

### HiFi outperformed CLR in detecting potentially true-positive variants of ≥ 50 bp by 3.4-fold

The difference in variant detection rate between CLR and HiFi was more pronounced for indels ≥ 50 bp. Of the ≥ 50-bp founder deletions identified using the CLR assembly, 213 at least partially matched (57% in ALT1 and 67% in ALT2) and 128 (60%) perfectly matched between the two strains (Fig. 3C). Meanwhile, of the ≥ 50-bp founder deletions identified using the HiFi assembly, 412 at least partially matched (81% in ALT1 and 84% in ALT2) and 374 (91%) perfectly matched between the two strains. The HiFi assembly detected a significantly higher number of common founder variants than the CLR assembly (Fig. 3C). The proportion of fully matched deletions was lower for ≥ 50-bp deletions than 5–49-bp deletions (Fig. 3A).

Regarding insertions ≥ 50 bp, HiFi detected more common founder variants than CLR. The proportion of partially matched ≥ 50-bp insertions was also higher than that of similar-sized deletions, demonstrating that even with HiFi, the detection accuracy of insertions decreased in larger variants (Fig. 3C, D).

To summarize, these results demonstrated that the use of the HiFi assembly improved variant calling performance, especially for SV detection, allowing detection of a 2.9-fold higher number of deletions and 4.5-fold higher number of insertions than the CLR assembly. In addition, we were able to detect a significant number of true-positives with lengths ≥ 1 kb using the HiFi assembly, while true-positives of this length were rarely detected using the CLR assembly (Additional file 1: Fig. S3).

### HiFi-based common founder variants covered a high proportion of CLR-based variants

We analyzed the overlap between the two true-positive call sets that consisted of common founder variants that perfectly matched between the two strains and confirmed that approximately 84% of CLR-based variants were covered by HiFi-based variants, regardless of type and size (Fig. 3E). On the other hand, approximately 30%–51% of HiFi-based variants were covered by CLR-based variants, with the lowest proportion observed for ≥ 50-bp deletions. These results indicated that the HiFi assembly allowed detection of a significant number of false-negatives missed in the CLR assembly and most variants detected with the CLR assembly.

### More number of potential false-positive variants were identified using CLR than HiFi

As ALT1 and ALT2 contain variants acquired through common and strain-specific DNA damage events, acquired variants can be divided into the following three types according to their sources: acquired variants common in ALT1 and ALT2 that were generated by common DNA damage, acquired variants differing between ALT1 and ALT2 that were generated by strain-specific DNA damage, and acquired variants differing between ALT1 and ALT2 that were generated by false-positive variant calls. We indirectly estimated the false-positive detection rate of CLR and HiFi using the proportions of common and strain-specific acquired variants. In both strains, HiFi identified fewer acquired variants than CLR, with ALT1 containing 2677 and 1715 acquired variants detected using CLR and HiFi, respectively, and ALT2 containing 2245 and 1597 acquired variants detected using CLR and HiFi, respectively (Additional file 1: Table S4).

**Fig. 4:**
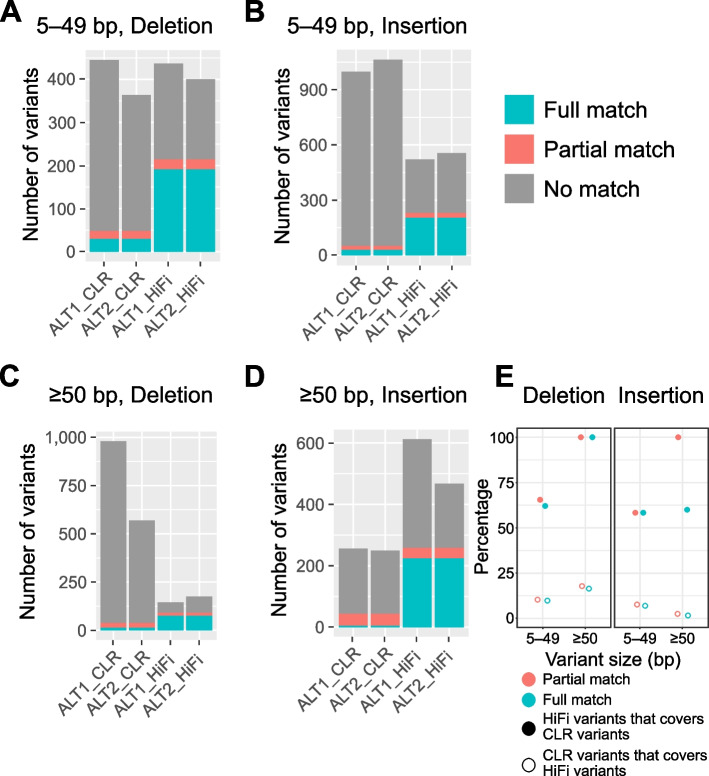
Acquired variant detection rates of CLR and HiFi assemblies to estimate relative false-positive calling rates. **A**–**D**, Number of shared or strain-specific acquired variants detected in each assembly. Acquired variants were divided into four groups according to their size and type: **A** 5–49-bp deletion; **B** 5–49-bp insertion; **C** ≥ 50-bp deletion; and **D** ≥ 50-bp insertion. Blue bar represents acquired variants perfectly matched for position and size between ALT1 and ALT2 using the same sequencing mode. Red bar represents partially-matched acquired variants. Gray bar represents the remaining acquired variants identified in a single strain. **E** Overlap ratios for common acquired variants detected using CLR and HiFi assemblies. Full circle represents the ratio of CLR variants covered by HiFi and the empty circle represents vice versa. Colors represent partial- or full-matched variant sets

Of the 5–49-bp deletions detected from the CLR assembly, only 48 (11% in ALT1 and 13% in ALT2) at least partially matched between ALT1 and ALT2, of which 29 (60%) perfectly matched for position and size (Fig. 4A). Among the 5–49-bp deletions detected from the HiFi assembly, 214 (50% and 54% in ALT1 and ALT2, respectively) at least partially matched between ALT1 and ALT2, of which 191 (89%) perfectly matched. In other words, 88% of the total acquired variants identified from the CLR assembly were not shared between the two strains, while < 50% of the total acquired variants identified from the HiFi assembly were shared between the two strains, indicating that some CLR-based variants may be false positives. The difference between the CLR and HiFi assemblies was more pronounced for 5–49-bp insertions. Based on the HiFi assembly, the proportion of insertions shared between the two strains was approximately 57%, while almost 95% of the variants identified from the CLR assembly were not shared between the two strains (Fig. 4B).

Similar results were observed for ≥ 50-bp indels. The HiFi assembly detected fewer deletions than the CLR assembly (979 for CLR and 145 for HiFi in ALT1 and 569 for CLR and 175 for HiFi in ALT2), of which 90 (57%) at least partially matched between the two strains. Among the variants identified in the CLR assembly, only 37 (5%) at least partially overlapped despite a sixfold higher number of ≥ 50-bp deletions being detected in the CLR assembly than in the HiFi assembly (Fig. 4C). In case of ≥ 50-bp insertions, several different variants between the two strains were detected based on CLR (Fig. 4D). As HiFi outperformed CLR in detecting common founder variants, these results indicate that the higher number of acquired variants detected using the CLR assembly is due to a substantial proportion of false positives, regardless of the variant length.

### HiFi-based common acquired variants covered a high proportion of CLR-based common acquired variants

While acquired variants may contain a large number of false positives, variants found in both ALT1 and ALT2 represent true positives shared between the two strains. We estimated the proportions of false positives detected using the CLR and HiFi assemblies by analyzing the ratio of common and different acquired variants captured using the two platforms, HiFi and CLR.

We analyzed the overlap between the common acquired variants detected using CLR and HiFi and confirmed that HiFi covered approximately 60% of the deletions and all insertions detected using CLR (Fig. 4E). However, the proportion of HiFi-based variants covered by CLR-based variants was 2%–17%.

The acquired variants detected using the two platforms did not overlap well. Regardless of the type and size of the variant, HiFi covered < 22% of the CLR-based variants, and CLR covered < 25% of the HiFi-based variants (Additional file 1: Fig. S4). We anticipated that HiFi would cover approximately 60% of CLR-based variants, as observed for common acquired variants. We hypothesized that the reduced coverage from 60% to 20% may be attributable to the threefold increase in the total number of variants due to miscalled false-positive variants in the CLR assembly. Therefore, we determined that up to two-thirds of all acquired variants in the CLR assembly were false-positive variants.

### 10 × HiFi sequencing data were sufficient to detect variants

Considering the higher cost of HiFi than CLR or short-read sequencing technologies, we hypothesized that reliable variant calling could be achieved with the use of smaller HiFi datasets. We utilized the common founder variants in ALT1 and ALT2 described above (1804 in total) as the true-positive variant set. We compared the read-based and assembly-based variant calling methods in combination with subsampling of various sequencing depths. We performed assembly-based variant calling with randomly extracted 5 × , 10 × , and 15 × HiFi data and read-based variant calling with HiFi read depths of 1 × to 15 × at 1 × intervals. For assembly-based variant calling, we used SVIM-asm [26]. For read-based variant calling, we tested four publicly available SV callers, SVIM [27], Sniffles [28, 29], PBSV (http://github.com/PacificBiosciences/pbsv), and MAMnet [30] (http://github.com/micahvista/MAMnet), in terms of their recall performance. MAMnet and SVIM outperformed the other two SV callers, and SVIM exhibited slightly higher and less variable detection rate for almost all the cases than MAMnet (Additional file 1: Fig. S5). We used SVIM for further analysis.

**Fig. 5:**
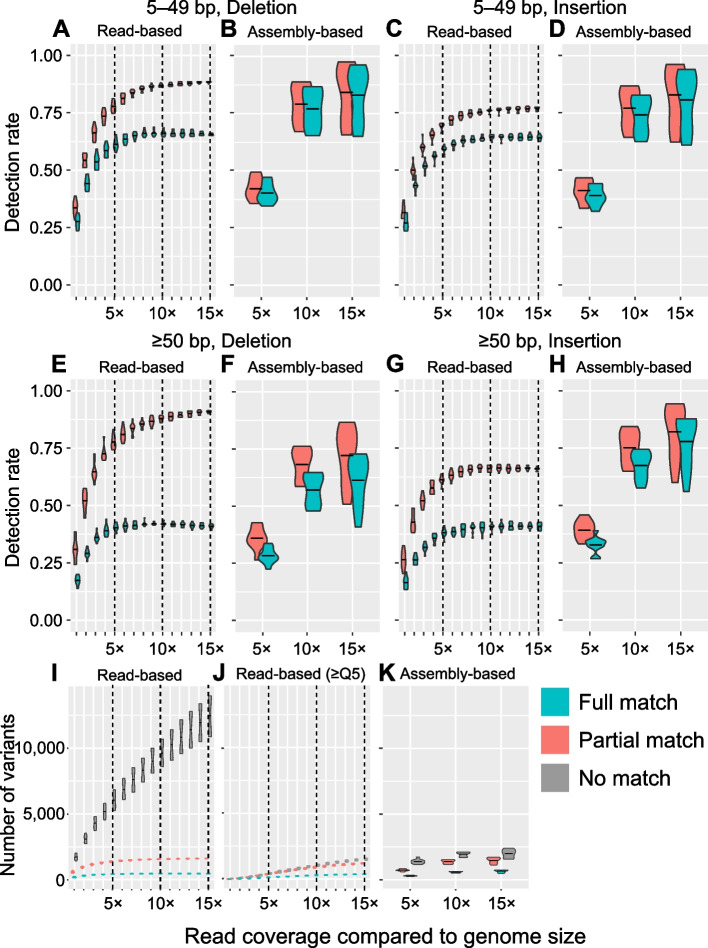
Performance of HiFi read- or assembly-based variant calling at varying sequencing depths. **A**–**H** Violin plots representing the detection rate of true-positive variants. We used the full-matched common founder variants in ALT1 and ALT2 described above (1804 in total) as the true-positive variant set to estimate the true-positive variant detection rate. Red represents the detection rate based on at least partial matching to the true-positive variant set and blue represents the detection rate based on perfect matching. **I**–**K** Violin plots representing the number of total detected variants. Red represents the number of variants at least partially matched to the common founder variants (1804 in total) and blue represents the detection rate based on perfect matching. Gray represents the remaining variants. Reliable variants were filtered from those obtained using read-based variant calling according to their quality metric (Q ≥ 5; Q5). Subsampling was repeated five times for each sequencing depth. Each horizontal solid line of the violin plot represents the median of five repeats

In all cases, greater amounts of data increased the detection rate of common founder variants, with both read-based and assembly-based variant calling methods and the detection rate was saturated at 10 × (Fig. 5). In read-based variant calling, the detection rates of deletions were higher than those of insertions (Fig. 5A, C, E, G), since insertions can be detected only when the corresponding reads are fully mapped to the reference, making calling of insertions more difficult than deletions. For variants that fully matched to the true-positive set, we confirmed that the detection rates of indels converged to 65% for 5–49-bp variants and 40% for ≥ 50-bp variants (Fig. 5A, C, E, G).

In contrast to read-based variant calling, in assembly-based variant calling, the detection rate for fully matched variants did not differ considerably from the detection rate for partially matched variants. The high quality of assembly-based variants may result from error correction during genome assembly. Low-depth assembly-based variant calling datasets typically have lower detection rates than depth-matched read-based variant calling datasets as depths lower than 10 × are too sparse to assemble the genome accurately. However, 10 × assembly-based variant calling had a higher insertion detection rate and accuracy (Fig. 5C, D, G, H) than 15 × read-based variant calling.

Taken together, these results demonstrated that the use of 10 × HiFi data is sufficient for successful variant calling, provided a good-quality reference is used. In addition, most variants can be accurately identified using assembly-based variant calling.

### Read-based variants contained poor relevant and possible false positives

Next, we confirmed whether the false-positive detection rate can be improved through genome assembly. We compared the total number of variants and common founder variants using read- and assembly-based variant calling data. In read-based variant calling, the number of common founder variants converged to 1600 above 10 × depth, but the number of variants that did not match common founder variants steadily increased and occupied up to 88% of all variants (Fig. 5I). Assembly-based variant calling showed substantially different results. The number of common founder variants converged to 1400 at depths > 10 × , but the number of variants that did not match common founder variants remained constant at around 1900, regardless of the depth. Based on these results, we hypothesized that substantially more false-positive variants resided in non-matched read-based variants than assembly-based.

To support this hypothesis, we filtered the read-based variants using their variant quality metric to designate reliable variants (Q ≥ 5; Q5). Among the total variants, only 20% remained after quality filtering, but still 60%–80% common founder variants (true-positive set) remained. Impressively, this filtering was much more powerful for non-matched variants of which only 10% remained (Fig. 5J). This result suggests that most of the variants detected using read-based variant calling may not be reliable although the total number of detected variants increase. In addition, even true-positive variants may not be well supported by statistical analyses: 10 × read-based reliable variant calling detected only 50% of the true-positive deletions and 40% of the true-positive insertions (Additional file 1: Fig. S6A–D). After this filtering, 10 × assembly-based variant calling had a higher detection rate than 15 × read-based reliable variant calling. These results indicate that genome assembly prior to variant calling is more reliable, even at low depths.

The other three read-based SV callers, Sniffles, PBSV, and MAMnet, showed poorer performance than SVIM in reliable variant calling (Additional file 1: Fig. S7). Sniffles that contained reliable SVs only (Q ≥ 25) captured ~ 1000 common founder variants with ≥ 10 × reads. However, it also contained ~ 5000 non-matched variants, which exceeds the number of SVIM (Q ≥ 5). PBSV had the least number of variants (~ 1500 SVs with 15 × reads), and captured only ~ 650 common founder variants. MAMnet had ~ 1000 reliable variants (Q ≥ 5), but captured ~ 530 common founder variants. Thus, SVIM exhibited better recall and precision performances than other read-based SV callers. However, as its sister assembly-based SV caller, SVIM-asm, outperformed SVIM, we recommend assembly-based SV calling rather than read-based one even with lower read depths.

## Discussion

Genetic variation is the fundamental basis of phenotypic variation, including human genetic diseases [31, 32] and evolution [33]. Despite ongoing efforts, technical limitations have hindered accurate calling of all genetic variants in populations [6, 7]. Recent innovations in long-read sequencing technologies have helped in overcoming these limitations by providing methods for the generation of high-quality genomes and identification of extremely large variants that were technically challenging using short-read sequencing technologies [15, 34, 35]. However, as long-read sequencing technologies have matured and several different methods are now available, there is a need for developing benchmarking analysis to compare sequencing technologies [13]. We therefore produced reads using HiFi, the most advanced accurate long-read sequencing technique, and compared the performance of HiFi and CLR, a noisy long-read sequencing technology. Our results may facilitate the development of methods for population-level variant calling, particularly construction of graph-based reference genomes.

The two *C*. *elegans* strains used in the present study exhibited two advantages in measuring variant calling metrics. First, as high-quality reference genomes are available that constitute the starting worm’s genome, we were able to accurately identify potential true-positive variants by utilizing the fact that they are inherited from a single starting worm and must be shared between the two strains. Second, the two strains contained variants acquired through common and strain-specific DNA damage that should be detected in a similar fashion by the same sequencing technology. Based on this assumption, we were able to estimate the proportions of false-positive variants detected using CLR and HiFi by obtaining common acquired variant ratios and comparing them to strain-specific variant ratios. As the variants of the two strains used do not require manual validation, they can be used to measure the performance of sequencing methods developed in the near future.

The limitations of the present study are related to the characteristics of *C*. *elegans*. *C*. *elegans* has a small genome size that allows generation of sufficient PacBio HiFi data at a lower cost than human samples; however, the genomic architecture of *C. elegans* is different from that of humans, with fewer repetitive elements and no centromeric regions [36, 37]. These characteristics may contribute to different performance metric values obtained when the same method is applied to human genomes. In addition, unlike humans, there is currently no *C*. *elegans* reference genome based on HiFi. Accordingly, we were unable to obtain the exact reference sequences of repetitive regions comparable to our HiFi data. Therefore, we were unable to evaluate the utility of HiFi in detecting variants in long-tandem repeat regions or accurately detect variants in the ribosomal RNA region. In addition, *C*. *elegans* is a selfing and highly homozygous hermaphrodite despite having diploid chromosomes [38]. Each of these factors may limit the direct application of our results to humans.

Another limitation was the lower throughput of HiFi sequencing than expected, with only 6 Gb of HiFi data on one cell of the PacBio Sequel II system. This result differs from that of previous studies in which 20–40 Gb data were generated on a single cell [39, 40]. This issue may be attributable to poor library preparation efficiency during the multiplexing process. It is important to confirm whether the low throughput observed in the present study was due to large deviations within the HiFi sequencing data or an unoptimized sequencing process at the facility that we used. In addition, as the depth of our HiFi reads was 20 × and the N50 lengths of HiFi assemblies were approximately 1 Mb, true- and false-positive variants present in loci that we failed to assemble were likely missed. The final limitation of the present study was that we were unable to compare the performance of variant calling among various sequencing technologies, such as short-read and Oxford Nanopore Technologies’ long- or ultra long-read sequencing technologies [41]. We expect to overcome these limitations by applying various sequencing technologies in the future.

Despite these limitations, the results of the present study demonstrated that accurate variant detection is possible using HiFi sequencing, the most advanced long-read sequencing technology. In particular, we were able to demonstrate that HiFi assembly-based variant detection is effective for the detection of long insertions, which is technically challenging with HiFi reads, and that 20 × HiFi data are sufficient for high-accuracy genome assembly. Furthermore, 10 × HiFi assembly was sufficient for high-quality indel detection. We expect that these methods can be directly applied to other species, such as *A. thaliana* [42] and *D. melanogaster* [43]*,* which have genome sizes comparable to *C. elegans*.

Variant detection based on accurate long-read sequencing is likely to become routine in the future, with advances toward high throughput and low cost. Population-level studies based on these advances will allow identification of valuable variants that have not been detected previously. We anticipate that tens of thousands of genome assemblies with reference quality, rather than whole genome sequencing data alone, will provide a fundamental basis for understanding the genetic architecture underlying phenotypic variation, thereby elucidating the “dark matter” in genomes.

## Conclusions

By directly comparing accurate and noisy long-read sequencing technologies, the results of the present study demonstrate that improvements in true- and false-positive variant calling can be achieved using HiFi instead of CLR. Furthermore, we present a HiFi experimental design that allows cost-effective and high-quality variant calling with consideration of the high cost per base pair of HiFi. Our findings also demonstrate that 10 × HiFi data are sufficient for efficient variant detection and that genome assembly is recommended prior to variant calling, even at low depths. The findings of the present study may facilitate the development of methods for detecting genetic variants at the population level.

## Methods

### *C*. *elegans* strains and accession numbers

All worms were derived from a telomerase mutant, *trt-1(ok410)*. CB4856 worms were maintained at 20 °C on nematode growth media (NGM) plates seeded with *E*. *coli* OP50. All strains used for sequencing in the present study were inbred by selfing twice. Public genome assemblies were used as reference genomes. The CB4856 genome (ASM452629v1) was downloaded from NCBI and the N2 genome (WBcel235/ce11) was downloaded from Ensembl (release 103).

#### Genomic DNA extraction and PacBio sequencing of multiplexed samples

Genomic DNA from each strain was extracted according to previously reported methods [20]. Multiplexed DNA sequencing library construction, sequencing, and demultiplexing were performed at Macrogen. Multiplexed libraries that were composed of ~ 20-kb insert molecules were sequenced to an average of 17 passes using the PacBio Single Molecule, Real-Time (SMRT) DNA sequencing technology (platform: PacBio Sequel II; mode: circular consensus sequencing mode). HiFi reads were automatically generated using SMRT link v10.1 with the default option, filtered according to the quality value (Q ≥ 20; Q20).

#### Genome assembly and quality assessment

Each genome was de novo assembled using HiFi reads with 21 × depth for ALT1 and 18 × depth for ALT2 using HiCanu (Canu version 2.0; *canu genomeSize*= *100m -pacbio-hifi)* [44]. ALT1 and ALT2 genome assemblies obtained using CLR are available under NCBI GenBank accessions, ASM1813687v1 and ASM1813680v1. The completeness of the assemblies based on CLR and HiFi reads was evaluated using BUSCO (BUSCO version 4.0.6; *busco -m genome -l nematoda_odb10*) [45, 46], and the length of repetitive elements was estimated based on repeat-masked genomic sequences using RepeatMasker (version 4.1.0; *RepeatMasker -species metazoa -s*).

### Identification of variants

For read-based variant calling, we used HiFi reads to detect genetic variants using the four SV calling methods, SVIM [27], Sniffles [28, 29], PBSV (http://github.com/PacificBiosciences/pbsv), and MAMnet [30] (http://github.com/micahvista/MAMnet). We used SVIM with its default parameters (version 1.4.2; *svim read --aligner minimap2 --min_sv_size 5 --skip_genotyping --minimum_depth 1*). For Sniffles, HiFi reads from each sample (N2, ALT1, and ALT2) were mapped to the CB4856 genome using minimap2 (version 2.22; *minimap2 -ax map-hifi*) [47], the output mapping files were sorted and indexed using SAMtools (version 1.13; *samtools sort -m4G -O BAM* and *samtools index*) [48], and these indexed BAM files were used to detect genetic variants (version 2.0.7; *sniffles -i --minsvlen 2 -v*). For PBSV, HiFi reads were mapped to the CB4856 genome using pbmm2 (version 1.10.0; *pbmm2 align --sort --preset CCS --sample --rg*) (https://github.com/PacificBiosciences/pbmm2) and the output BAM files were used to detect genetic variants (version 2.8.0; *pbsv discover* and *pbsv call --ccs*). For MAMnet, HiFi reads were mapped to the CB4856 genome using minimap2 (version 2.22; *minimap2 -ax map-hifi --MD*), the output mapping files were sorted and indexed using SAMtools (version 1.13; *samtools sort -m4G -O BAM* and *samtools index*), and these indexed BAM files were used to detect genetic variants (*python MAMnet.py -bamfilepath -workdir -outputpath*). For assembly-based variant calling, contigs from each assembly (N2, ALT1, and ALT2) were aligned to the CB4856 genome using minimap2 (version 2.22; *minimap2 -a -x asm5 --cs -r2k*) and the output alignment was sorted and indexed using SAMtools (version 1.13; *samtools sort -m4G -O BAM* and *samtools index*). These indexed BAM files were used to detect genetic variants using SVIM-asm (SVIM-asm version 1.0.2; *svim-asm haploid --min_sv_size 5*) [26].

#### Classification of founder and acquired variants and comparison between CLR and HiFi

Deletions and insertions were divided into the following two groups depending on the size of variants: 5–49 bp and ≥ 50 bp. We used different BEDTools options to categorize variants according to partial matching and full matching (version 2.30.0; *bedtools intersect -wa -a -b* for partially matched deletions and insertions; *bedtools intersect -wa -r -f 1.0 -a -b* and *bedtools intersect -wa -wb -r -f 1.0 -a -b | awk '$4* == *$8'* for fully matched deletions and insertions, respectively) [49]. In comparison with CB4856 as a reference genome, indels that matched at least partially between N2 and ALT1 or ALT2 were classified as founder variants. Other variants were categorized as acquired variants and analyzed using BEDTools with the same options. The commonality of variant sets was further analyzed based on overlaps between ALT1 and ALT2 using BEDtools with the same options.

#### Variant detection analysis of HiFi data at varying depths

Subsampling of HiFi reads was performed at depths from 1 × to 15 × at 1 × intervals and repeated five times for each depth using seqtk (version 1.3-r106; *seqtk sample*). Each set of subsampled HiFi reads was used to detect genetic variants using SVIM for read-based variant calling as described above. HiFi reads that were previously extracted at depths 5 × , 10 × , and 15 × depths using seqtk were de novo assembled using Hifiasm (version 0.15.4; *hifiasm -l0*) [50]. We used Hifiasm instead of HiCanu because HiCanu does not perform well when datasets with low depths are used. Each assembly was aligned to the reference genome using minimap2. Output alignment files were sorted and indexed using SAMtools and output BAM files were used to detect genetic variants using SVIM-asm as described above. The dataset of common founder variants between ALT1 and ALT2 (1804 in total) was used as a true-positive set. Detection rates for the true-positive set at each depth were estimated by comparing the true-positive set with indels detected from read-based or assembly-based variant calling using BEDtools as described earlier. Reliable variants were filtered according to the quality metric (Q ≥ 5; Q5) from read-based variant calling.

## Supplementary Information

**Additional file 1:** **Fig. S1.** Length distribution of HiFi reads for two multiplexed samples, ALT1 and ALT2 (shown in color). Each vertical dashed line represents the average read length for each sample. **Fig. S2.** Summary of assembly-based variant calling outputs using SVIM-asm. Variants are categorized into five types: DEL, deletion; INS, insertion; INV, inversion; DUP_INT, interspersed duplication; and DUP_TAN, tandem duplication. Each graph on the left shows variants <2 kb. Graphs on the right show all variants. Each table in the right graph presents the number of variants in the corresponding call set. **Fig. S3.** Size distribution of common founder variants shared between ALT1 and ALT2 detected using the CLR or HiFi assemblies. The numbers of variants ranging in size from 5 bp to ≤10 kb and >10 kb. The numbers of >10-kb variants were too small and are therefore represented as absolute numbers (0 or 1). **Fig. S4.** Overlap ratio between all acquired variants detected using CLR and HiFi for each strain. A, Proportion of CLR variants covered by HiFi. B, Proportion of HiFi variants covered by CLR. Color represents partial- or full-matched variant sets. Shapes (circle or triangle) represent each strain. **Fig. S5.** Performance of four read-based SV callers using HiFi reads in various sequencing depths. Violin plots represent the number of total detected variants. Violin plots represent the detection rate of true-positive variants from the four SV callers, SVIM, Sniffles, PBSV, and MAMnet. We used the full-matched common founder variants in ALT1 and ALT2 described above (total 1804) as the true-positive variant set to estimate the true-positive variant detection rate. Red represents the detection rate based on at least partial match to the true-positive variant set, and blue does only for perfect match. Subsampling was repeated 5 times for each sequencing depth. Each horizontal solid line of the violin plot means a median of each 5 repeats. **Fig. S6.** Performance of HiFi read-based reliable variant calling at varying sequencing depths. Violin plots representing the detection rate of true-positive variants. The full-matched common founder variants in ALT1 and ALT2 described above (1804 in total) were used as the true-positive variant set. Red represents the detection rate based on at least partial matching to the true-positive variant set and blue represents the detection rate based on perfect matching. Subsampling was repeated five times for each sequencing depth. Reliable variants were filtered from those obtained using read-based variant calling according to their quality metric (Q ≥ 5; Q5). **Fig. S7.** Performance of the three SV callers, Sniffles, PBSV, and MAMnet, in detecting reliable true-positive variants in various sequencing depths. Violin plots represent the number of total detected variants. Red represents the number of variants at least partially matched to the common founder variants (total 1804), and blue does only for perfect match. Gray represents the remaining variants out of the total. Reliable variants were filtered by their quality metric (Q ≥ 5; Q5) from read-based variant calling, except Sniffles and PBSV, in which the quality metric is over 25 or missing, respectively. Subsampling was repeated 5 times for each sequencing depth.** Table S1.** Statistics for polymerase reads and subreads. **Table S2.** Statistics for HiFi reads. **Table S3.** Statistics for assembled contigs built on CLR or depth-matched HiFi reads. **Table S4.** Number of founder and acquired variants detected in each assembly for each strain.

## Data Availability

The datasets supporting the conclusions of this article are available in the NCBI BioProject repository (https://www.ncbi.nlm.nih.gov/bioproject) under the accession number PRJNA896647 and in the Korean Nucleotide Archive (KoNA, https://kobic.re.kr/kona) under the accession number PRJKA220495 (sequencing data only).
