# Supplementary material for: Benchmarking datasets for assembly-based variant calling using high-fidelity long reads
Source: BMC Genomics. 2023 Mar 27;24:148. doi: 10.1186/s12864-023-09255-y (PMC10045170; doi:10.1186/s12864-023-09255-y)
Supplement: Supplementary file 1 — Additional file 1: Fig. S1. Length distribution of HiFi reads for two multiplexed samples, ALT1 and ALT2 (shown in color). Each vertical dashed line represents the average read length for each sample. Fig. S2. Summary of assembly-based variant calling outputs using SVIM-asm. Variants are categorized into five types: DEL, deletion; INS, insertion; INV, inversion; DUP_INT, interspersed duplication; and DUP_TAN, tandem duplication. Each graph on the left shows variants <2 kb. Graphs on the right show all variants. Each table in the right graph presents the number of variants in the corresponding call set. Fig. S3. Size distribution of common founder variants shared between ALT1 and ALT2 detected using the CLR or HiFi assemblies. The numbers of variants ranging in size from 5 bp to ≤10 kb and >10 kb. The numbers of >10-kb variants were too small and are therefore represented as absolute numbers (0 or 1). Fig. S4. Overlap ratio between all acquired variants detected using CLR and HiFi for each strain. A, Proportion of CLR variants covered by HiFi. B, Proportion of HiFi variants covered by CLR. Color represents partial- or full-matched variant sets. Shapes (circle or triangle) represent each strain. Fig. S5. Performance of four read-based SV callers using HiFi reads in various sequencing depths. Violin plots represent the number of total detected variants. Violin plots represent the detection rate of true-positive variants from the four SV callers, SVIM, Sniffles, PBSV, and MAMnet. We used the full-matched common founder variants in ALT1 and ALT2 described above (total 1804) as the true-positive variant set to estimate the true-positive variant detection rate. Red represents the detection rate based on at least partial match to the true-positive variant set, and blue does only for perfect match. Subsampling was repeated 5 times for each sequencing depth. Each horizontal solid line of the violin plot means a median of each 5 repeats. Fig. S6. Performance of HiFi read-based [file 12864_2023_9255_MOESM1_ESM.pdf]

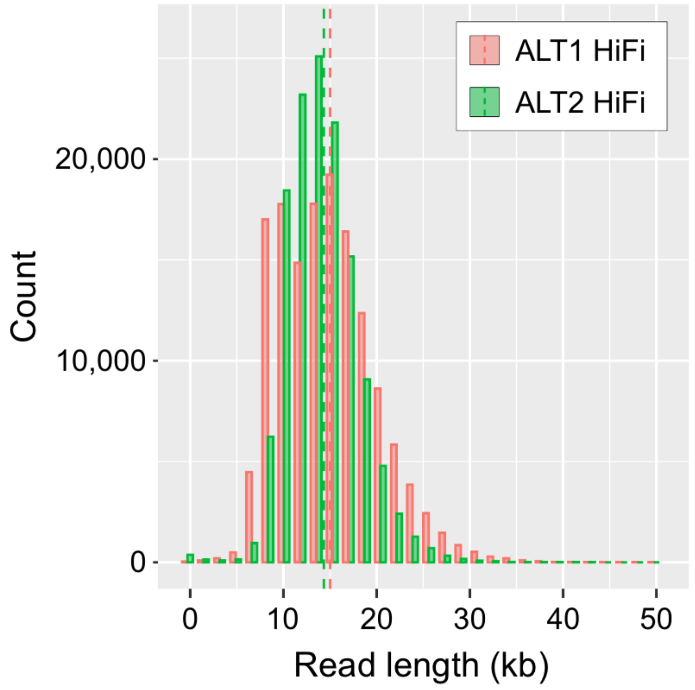

Fig. S1. Length distribution of HiFi reads for two multiplexed samples, ALT1 and ALT2 (shown in color). Each vertical dashed line represents the average read length for each sample.

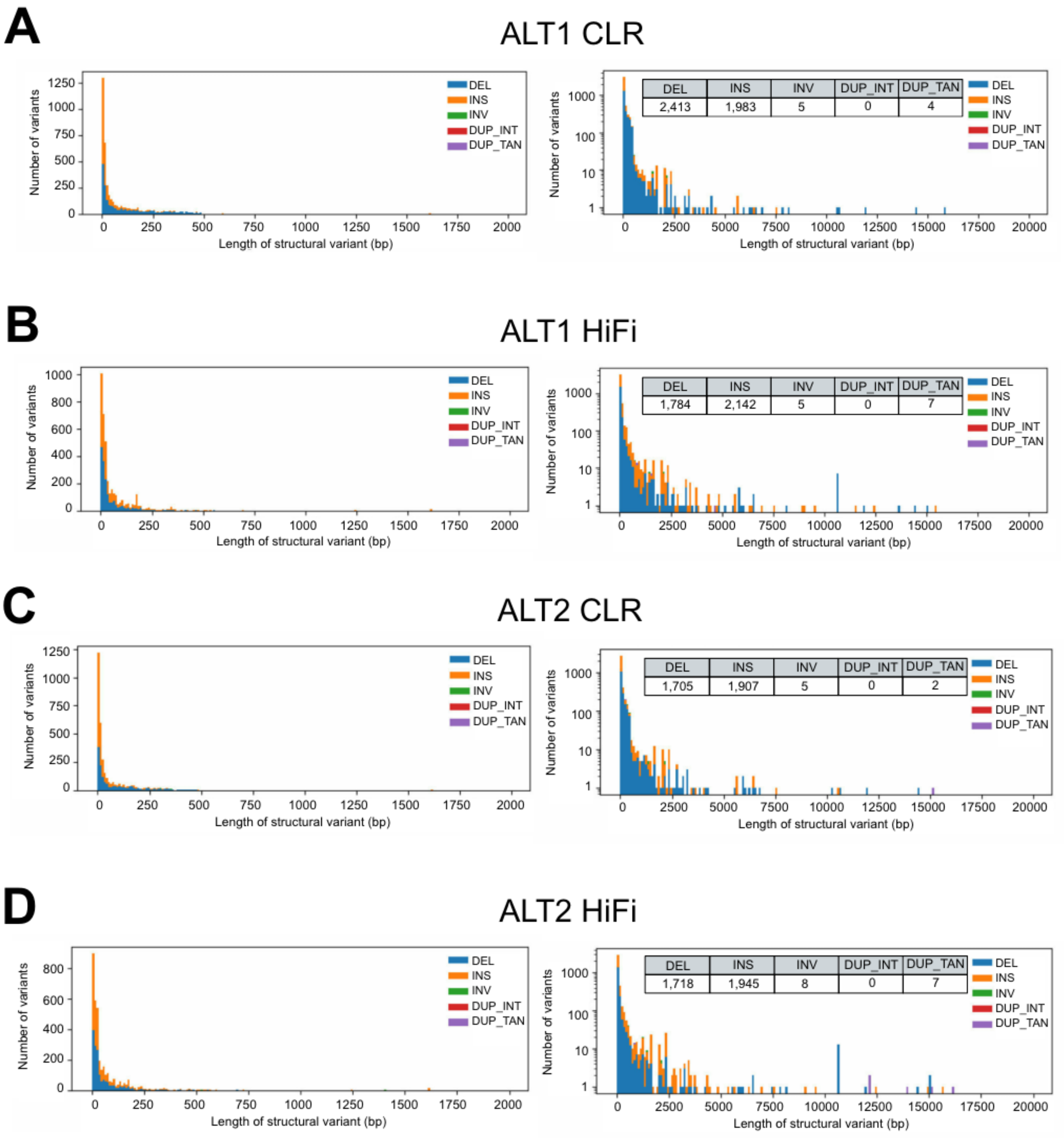

Fig. S2. Summary of assembly-based variant calling outputs using SVIM-asm.
Variants are categorized into five types: DEL, deletion; INS, insertion; INV, inversion; DUP_INT, interspersed duplication; and DUP_TAN, tandem duplication. Each graph on the left shows variants <2 kb. Graphs on the right show all variants. Each table in the right graph presents the number of variants in the corresponding call set.

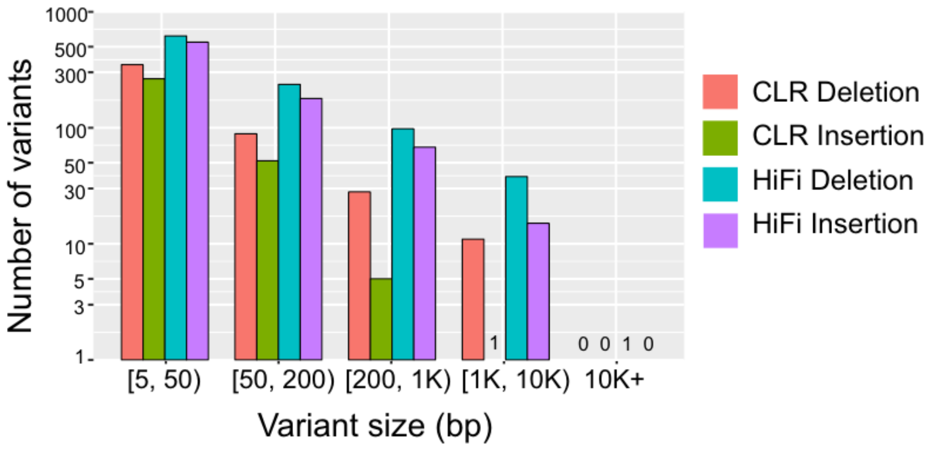

Fig. S3. Size distribution of common founder variants shared between ALT1 and ALT2 detected using the CLR or HiFi assemblies.

The numbers of variants ranging in size from 5 bp to ≤10 kb and >10 kb. The numbers of >10-kb variants were too small and are therefore represented as absolute numbers (0 or 1).

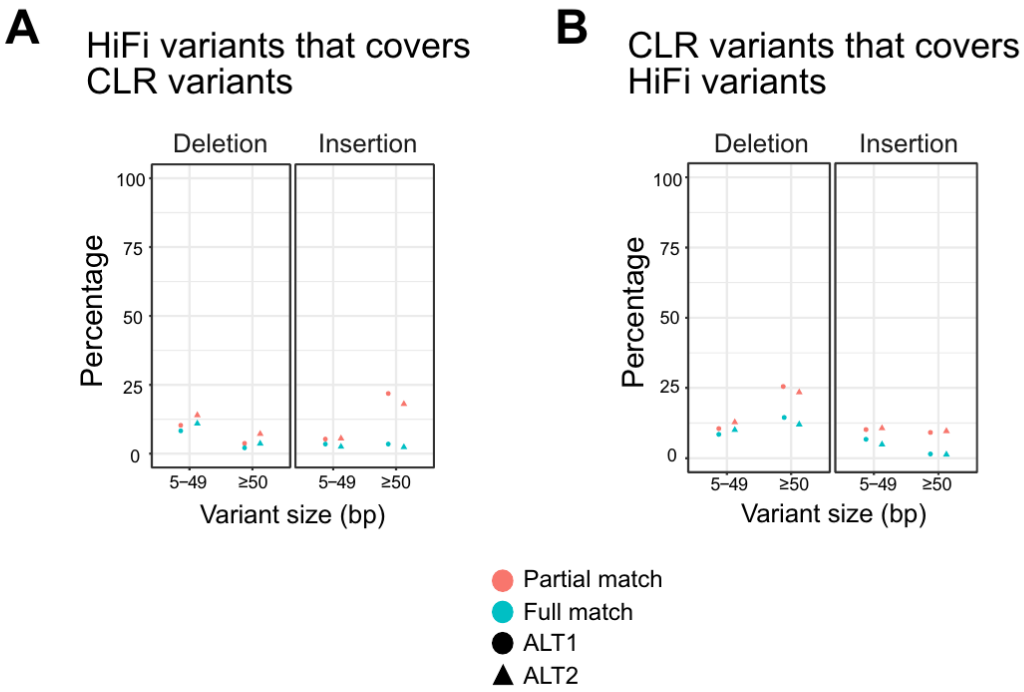

Fig. S4. Overlap ratio between all acquired variants detected using CLR and HiFi for each strain.

A, Proportion of CLR variants covered by HiFi. B, Proportion of HiFi variants covered by CLR. Color represents partial- or full-matched variant sets. Shapes (circle or triangle) represent each strain.

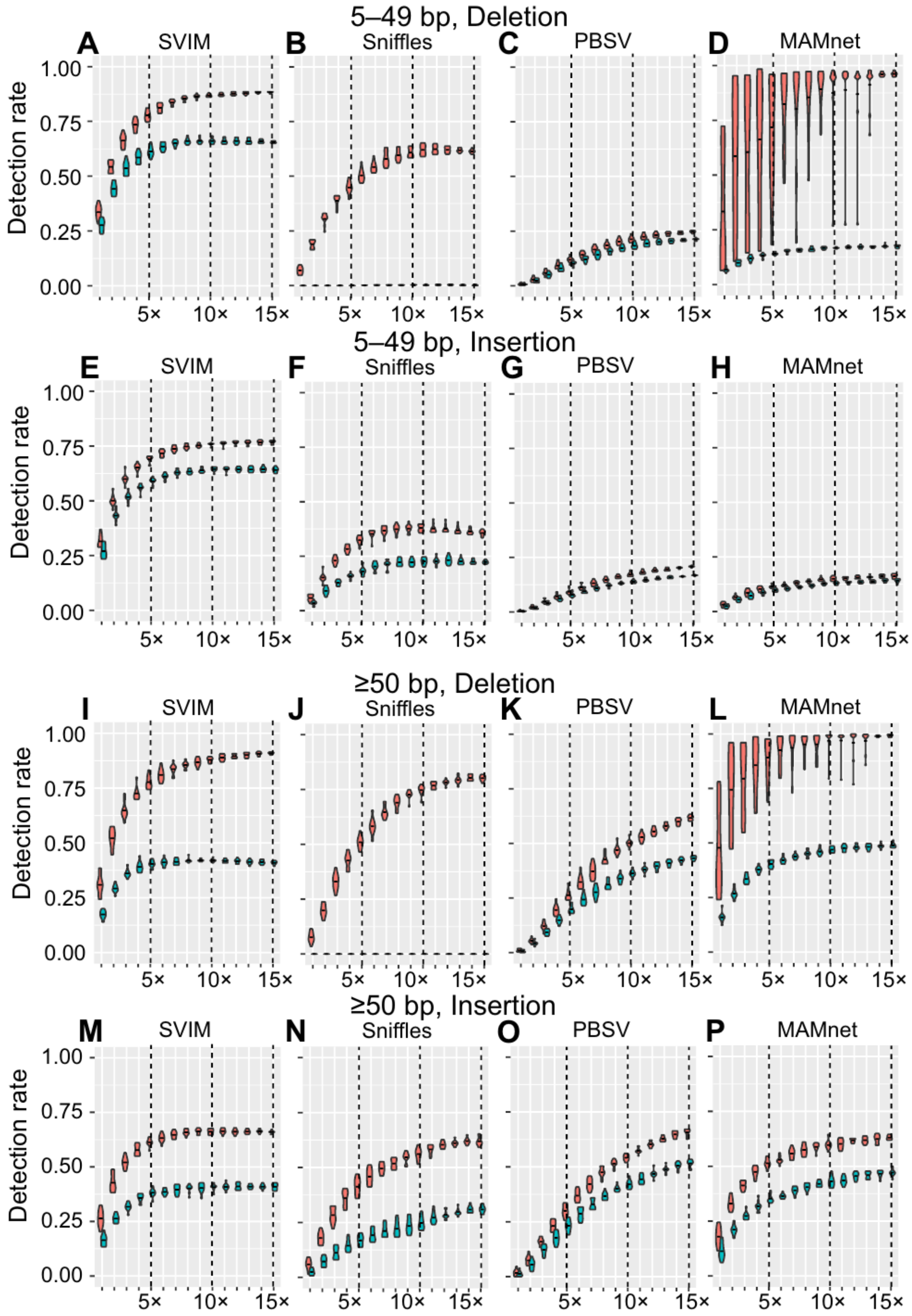

Fig. S5. Performance of four read-based SV callers using HiFi reads in various sequencing depths.

Violin plots represent the number of total detected variants. Violin plots represent the detection rate of true-positive variants from the four SV callers, SVIM, Sniffles, PBSV, and MAMnet. We used the full-matched common founder variants in ALT1 and ALT2 described above (total 1804) as the true-positive variant set to estimate the true-positive variant detection rate. Red represents the detection rate based on at least partial match to the true-positive variant set, and blue does only for perfect match. Subsampling was repeated 5 times for each sequencing depth. Each horizontal solid line of the violin plot means a median of each 5 repeats.

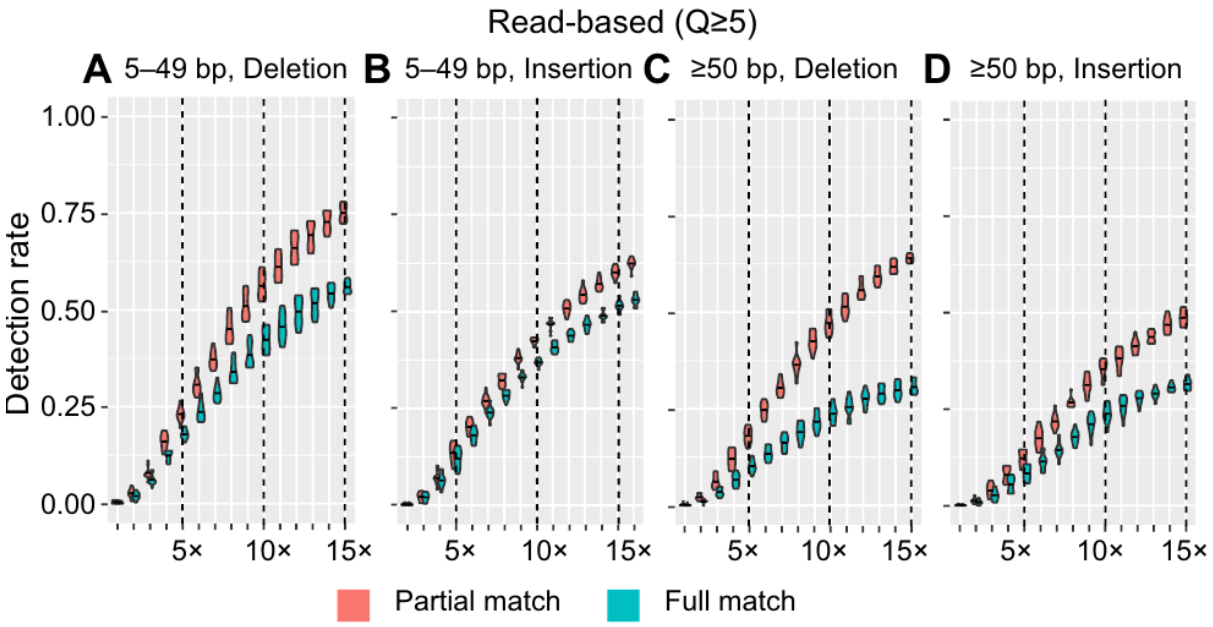

Fig. S6. Performance of HiFi read-based reliable variant calling at varying sequencing depths.

Violin plots representing the detection rate of true-positive variants. The full-matched common founder variants in ALT1 and ALT2 described above (1804 in total) were used as the true-positive variant set. Red represents the detection rate based on at least partial matching to the true-positive variant set and blue represents the detection rate based on perfect matching. Subsampling was repeated five times for each sequencing depth. Reliable variants were filtered from those obtained using read-based variant calling according to their quality metric (Q ≥ 5; Q5).

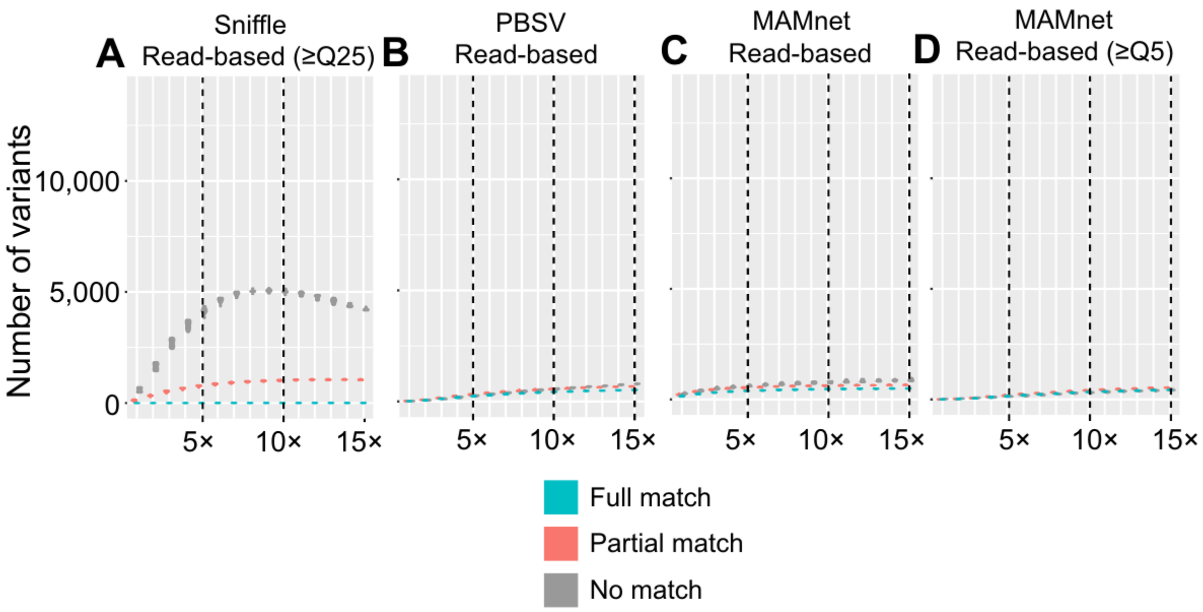

Fig. S7. Performance of the three SV callers, Sniffles, PBSV, and MAMnet, in detecting reliable true-positive variants in various sequencing depths.
Violin plots represent the number of total detected variants. Red represents the number of variants at least partially matched to the common founder variants (total 1804), and blue does only for perfect match. Gray represents the remaining variants out of the total. Reliable variants were filtered by their quality metric (Q ≥ 5; Q5) from read-based variant calling, except Sniffles and PBSV, in which the quality metric is over 25 or missing, respectively. Subsampling was repeated 5 times for each sequencing depth.

Table S1. Statistics for polymerase reads and subreads.

Sample	Number of polymerase read bases	Number of polymerase reads	Polymerase read N50 (kb)	Average read length (kb)
Total	99,785,359,907	1,965,091	150.583	50.779
**Sample**	**Number of subread bases**	**Number of subreads**	**Subread N50 (kb)**	**Average read length (kb)**
ALT1	30,437,031,725	2,353,947	15.551	12.930
ALT2	27,654,170,968	2,475,522	14.526	11.171
ALT5	27,756,282,994	2,417,821	14.214	11.479

Statistics for pooled polymerase reads from the three samples and the subreads of each sample generated after demultiplexing. We used only two samples, ALT1 and ALT2, out of the three samples.

Table S2. Statistics for HiFi reads.

Sample	Total size (Mb)	Number of reads	N50 (kb)	Max (kb)	Average (kb)	Depth (X)
ALT1	2,175	144,932	16.370	50.068	15.01	21.75
ALT2	1,872	130,547	14.924	43.253	14.345	18.72
ALT5	1,863	130,761	14.657	46.493	14.251	18.63

Table S3. Statistics for assembled contigs built on CLR or depth-matched HiFi reads.

Sample	Reads	Total size (Mb)	Number of contigs	N50 (kb)	Max (Mb)	Average (kb)	BUSCO genes (%)
ALT1	CLR	104.56	763	388.79	1.47	137.04	85.0
	HiFi	107.05	336	1228.12	5.04	318.61	97.5
ALT2	CLR	101.86	754	389.85	2.08	135.09	78.8
	HiFi	106.97	439	968.24	5.10	243.66	94.5

Table S4. Number of founder and acquired variants detected in each assembly for each strain.

Sample	Platform	Number of total indels	Number of founder variants			Number of acquired variants		
			Total	5-49 bp	≥50 bp	Total	5-49 bp	≥50 bp
ALT1	CLR	4396	1719	1135	584	2677	1442	1235
	HiFi	3926	2211	1372	839	1715	958	757
ALT2	CLR	3657	1412	918	494	2245	1427	818
	HiFi	3663	2066	1271	795	1597	955	642

The total indels of each strain represent all deletions and insertions ≥5 bp detected via SVIM-asm using the CB4856 genome as a reference. Total indels were classified into two groups, founder or acquired variants, depending on whether each variant had at least partial overlap with the known N2-type variants or not, respectively.
